# Early drainage reduces the length of hospital stay in patients with lung abscess

**DOI:** 10.3389/fmed.2023.1206419

**Published:** 2023-09-05

**Authors:** Po-Chang Chiang, Chia-Ying Lin, Ya-Chun Hsu, Li-Ting Huang, Ta-Jung Chung, Yi-Sheng Liu, Chao-Chun Chang

**Affiliations:** ^1^Department of Medical Imaging, National Cheng Kung University Hospital, College of Medicine, National Cheng Kung University, Tainan, Taiwan; ^2^Department of Surgery, Division of Thoracic Surgery, National Cheng Kung University Hospital, College of Medicine, National Cheng Kung University, Tainan, Taiwan

**Keywords:** lung abscess, drainage, interventional radiology (IR), length of stay, treatment outcome

## Abstract

**Background:**

Although percutaneous transthoracic catheter drainage (PCD) has been proven effective in lung abscesses, the optimal timing of PCD is still unclear. The study aimed to evaluate the safety and efficacy of early versus delayed drainage in patients with lung abscesses.

**Methods:**

This retrospective study included 103 consecutive patients with liquefied lung abscesses more than 3 cm confirmed by a CT scan received CT-guided PCD over 16 years, from July 2005 to September 2021, in a single institution were reviewed. Early drainage was defined as PCD within one week after a lung abscess was diagnosed. The primary outcome was 90-day mortality. The secondary outcomes included perioperative complications and patients’ length of hospital stay (LoS). Factors associated with 90-day mortality and LoS were also analyzed. The key statistical methods were Chi-square test, Fisher’s exact test, Student *t*-test, and Pearson correlation.

**Results:**

Amount the 103 patients, there were 64 patients who received early PCD, and 39 patients received delayed PCD. Between the two groups, there were no significant differences in clinical characteristics, 90-day mortality, or perioperative complications. The LoS was significantly shortened in early PCD group (28.6 ± 25.5 vs. 39.3 ± 26.8 (days), *p* = 0.045). Higher Charlson comorbidity index, secondary lung abscess, and liver cirrhosis were associated with higher mortality (all *p* < 0.05). Positive sputum culture significantly increased the LoS (coefficient 19.35 (10.19, 28.50), *p* < 0.001).

**Conclusion:**

The 90-day mortality and complications were similar for early PCD and delayed PCD patients, but LoS was significantly shortened in early PCD patient.

## 1. Introduction

Lung abscess was once a life-threatening disease, with a mortality rate of up to 75% (1). The outcomes of lung abscesses have improved over past decades due to timely diagnosis, effective antimicrobial therapy, application of drainage, or surgery. Currently, about 80%–90% of pyogenic lung abscesses are treated successfully with antibiotics. However, 10%–20% of patients with lung abscesses need invasive treatment, such as surgical intervention or percutaneous transthoracic catheter drainage (PCD), due to conservative treatment failure. In the past, PCD for lung abscesses was usually reserved for patients who fail to improve after antibiotic treatment (2–7). Because of the concerns about major complications, such as bronchopleural fistula (BPF), pneumothorax, hemoptysis, and empyema (8). With technological improvement, image-guided PCD is considered less invasive and safer than surgical intervention (9, 10). It can also bridge the gap between medical and operative management (11). The role of early PCD remains unclear. Current evidence, comprising a single or a few case series, show a high success rate without major complication or mortality in early PCD with concurrent antibiotics (12–14). Given the paucity of data in this field, we aim to compare the safety and efficacy of early versus delayed drainage in patients with lung abscesses. We hypothesize that early PCD results in better outcomes compared with delayed PCD.

## 2. Materials and methods

### 2.1. Study population

**Figure 1:**
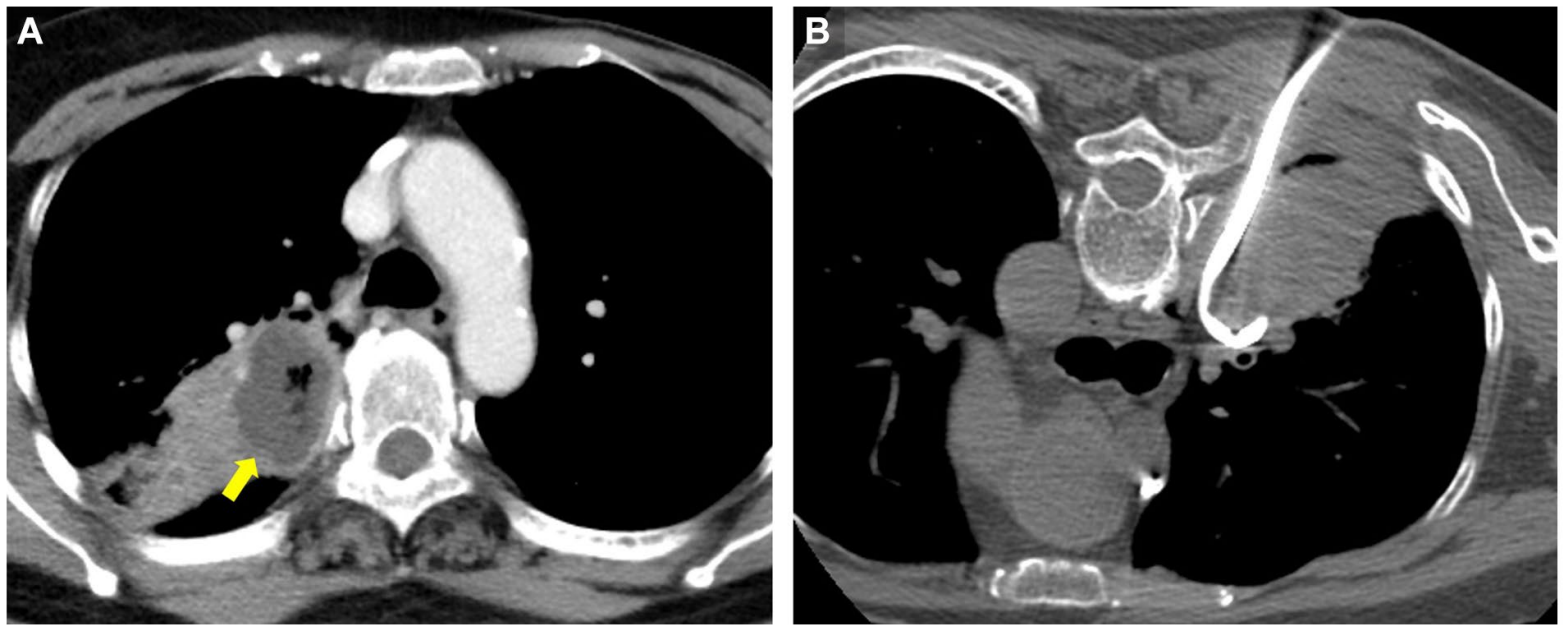
Representative contrast-enhanced CT (CECT) images with liquefied lung abscess in a 69 years-old woman who presented with fever and cough for 2 weeks. **(A)** CECT shows a 3.7 cm low-attenuation necrotic region (arrow) with gas formation. **(B)** CT-guided PCD was performed after CECT at the same day.

This retrospective study was approved by the Institutional Review Board of the National Cheng Kung University Hospital (A-ER-111-325), and informed consent was waived. Between July 2005 and September 2021, all patients who underwent CT-guided PCD for lung abscesses at National Cheng Kung University were reviewed. The diagnosis of lung abscess was based on CT imaging findings and clinical presentation. Criteria for CT-guided drainage included liquefied abscess greater than 3 cm (Figure 1) with ongoing sepsis. Exclusion criteria included incomplete clinical or radiological data.

### 2.2. Data collection and definitions

Age, sex, size of the abscess, white blood cell (WBC) count, C-reactive protein (CRP), cause of lung abscesses, Charlson comorbidity index, underlying diseases or malignancy, presence of preprocedural emphysema or pulmonary embolism, duration of pre-drainage antibiotics, the size of the drainage tube, whether trans-aerated lung parenchyma or not, drainage tube indwelling time, microbiological results, rationales of antibiotic use, subsequent surgery were recorded. The abscess size was determined by measuring the maximum diameter of the largest abscess identified. The duration of pre-drainage antibiotics was defined as the time interval between the first dose of intravenous antibiotics and the day of CT-guided PCD. There were primary and secondary causes of lung abscesses. Primary abscesses were infectious in origin, caused by aspiration or pneumonia in the healthy host. Secondary abscesses were caused by a preexisting condition (e.g., obstruction), spread from an extrapulmonary site, or bronchiectasis. The rationales for antibiotic use included empirical and culture-based. The subsequent surgery included lung resection and decortication.

Early PCDs were defined as PCDs performed within 1 week (≦7 days) after a lung abscess was diagnosed. Delayed PCDs meant PCDs were performed more than 1 week (>7 days) after a lung abscess was diagnosed. In general, PCDs were kept in place for at least 2 weeks before removal. If there were persistent air leaks from the drainage tube, the drainage tube was kept in place for 3 weeks to form a bronchopleurocutaneous fistula. The wound was compressed by sterile gauze after drainage tube removal. Reasons for drainage removal included recovery, dislodgement, subsequent surgery, or death.

### 2.3. CT-guided drainage technique

The drainage procedures were performed by a total of 19 board-certificated radiologists during the study period, with or without assistance from a resident. Drainage was performed using standard sterile technique with CT guidance (Optima 660, General Electric) using the Seldinger technique and local anesthesia. The size of the catheter and the route of placement were determined based on the cavity size and clinical experience of the attending radiologists. The abscess contents were aspirated, and the pus was sent for microbiologic culture. In general, empiric antibiotics were prescribed before PCD, and specific antibiotics were given once the culture results were available. Post-procedure imaging was performed to ensure the proper positioning of the drainage catheter. Abnormal coagulation profiles, including INR above 1.5 or platelets below 50,000/mL, were corrected before the procedure. If there were pre-procedure empyema, another pleural drainage tube was inserted.

### 2.4. Outcomes

The primary outcome was postoperative mortality within 90 days. Secondary outcomes included perioperative complications and patients’ length of hospital stay (LoS). Perioperative complications included BPF, empyema, hemoptysis, and pneumothorax.

### 2.5. Statistical analysis

Patient demographics and lesion characteristics were summarized using descriptive statistics (mean ± standard deviation for continuous variables and proportions for categorical variables). Differences between categorical and continuous variables between the two groups were compared using the chi-square test or Fisher’s exact test and student *t*-test, respectively. Correlations between continuous variables were analyzed using Pearson correlation. A *p* < 0.05 was set to indicate statistical significance. SPSS system (IBM SPSS Statistics, Version 22.0, Armonk, NY) was used for statistical analysis.

## 3. Results

### 3.1. Basic characteristics

**Figure 2:**
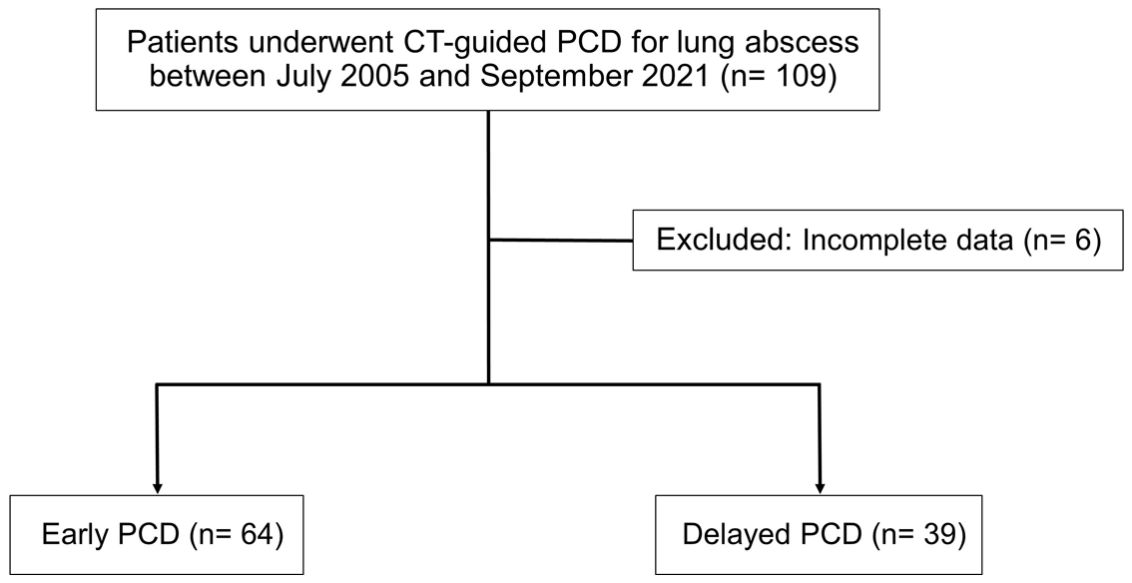
Flowchart outlining the selection of study subjects.

The study flowchart is presented in Figure 2. Among 109 patients who received PCD between July 2005 and September 2021, 6 were excluded due to missing clinical or radiographic data. The final study consisted of 103 patients: 64 received early PCD (mean age 59.2 years ±20.7), and 39 received delayed PCD (mean age 65.3 years ±14.6). Demographic characteristics are summarized in Table 1 and detailed in Supplementary Table S1. The patient demographics and clinical characteristics, including age, sex, size of the abscess, WBC, CRP, cause, Charlson comorbidity index, underlying disease, pre-procedure empyema, pulmonary embolism, drainage tube size, the distance of trans-aerated lung parenchyma, drainage tube indwelling time, culture positivity, the rationale of antibiotics use, were similar between the two groups. The overall positivity rates were 77.7% (80/103) from abscess culture, 45.6% (47/103) from sputum culture, and 3.9% (4/103) from blood culture. A total of 15 patients received subsequent surgery after PCD treatment failure, including 6 lung resections, 6 decortications, and 3 with both lung resection and decortication.

**Table 1:** Baseline characteristics of patients undergoing PCD for lung abscess.

Variables	Early PCD (*n* = 64)	Delayed PCD (*n* = 39)	*p*-value
Age (years)^a^	60.8 ± 18.9	59.8 ± 21.2	0.798
Sex (male, %)	51 (79.7%)	28 (71.8%)	0.358
Size of abscess (cm)^a^	7.5 ± 3.1	6.7 ± 2.4	0.199
WBC (× 10^9^/L)^a^	13.8 ± 8.0	13.8 ± 7.2	0.982
CRP (mg/dL)^a^	210.4 ± 103.0	141.8 ± 81.5	0.068
*Cause*			
Primary	33 (51.6%)	21 (53.8%)	0.822
Secondary	31 (48.4%)	18 (46.2%)	
Charlson comorbidity index^a^	5.8 ± 3.2	6.0 ± 3.8	0.845
*Underlying disease*			
Solid tumor	37 (57.8%)	21 (53.8%)	0.694
Lung cancer	19 (29.7%)	8 (20.5%)	0.304
Esophageal cancer	7 (10.9%)	4 (10.3%)	0.914
Other cancer	12 (18.8%)	11 (28.2%)	0.264
DM	24 (37.5%)	11 (28.2%)	0.334
CKD	8 (12.5%)	10 (25.6%)	0.088
Liver cirrhosis	6 (9.4%)	4 (10.3%)	1
Pre-procedure empyema	18 (28.1%)	11 (28.2%)	0.993
Pulmonary embolism	5 (7.8%)	3 (7.7%)	0.982
Tube size (Fr.)^a^	10.8 ± 2.7	10.2 ± 2.2	0.275
Distance of trans-aerated lung parenchyma (cm)	0.13 ± 0.41	0.09 ± 0.29	0.575
Drainage tube indwelling time (days)^a^	14.0 ± 15.6	25.6 ± 38.2	0.076
Positive abscess culture	53 (82.8%)	27 (69.2%)	0.108
Positive sputum culture	29 (45.3%)	18 (46.2%)	0.934
Positive blood culture	3 (4.7%)	1 (2.6%)	1
Antibiotics			0.597
Empirical	9 (14.1%)	7 (17.9%)	
Culture-based	55 (85.9%)	32 (82.1%)	
*Subsequent surgery*			
Lung resection	6 (9.4%)	3 (7.7%)	1
Decortication	4 (6.3%)	5 (12.8%)	0.294

Except where indicated, data are numbers of participants, with percentages in parenthesis.

a Data are mean ± standard deviation.

### 3.2. Outcomes

The primary and secondary outcomes are presented in Table 2. There was no significant difference in 90 days mortality between the two groups [early PCD group: 11 of 64 patients (17.2%), delayed PCD group: 10 of 39 patients (25.6%); *p* = 0.302]. The total complication was 8.7% (9/103). The two groups had a similar complication rate, included BPF [early PCD group: 1 of 64 patients (1.6%), delayed PCD group: 0 of 39 patients (0%); *p* = 1], empyema [early PCD group: 1 of 64 patients (1.6%), delayed PCD group: 3 of 39 patients (7.7%); *p* = 0.151], hemoptysis [early PCD group: 1 of 64 patients (1.6%), delayed PCD group: 0 of 39 patients (0%); *p* = 1], and pneumothorax [early PCD group: 2 of 64 patients (3.1%), delayed PCD group: 1 of 39 patients (2.6%); *p* = 0.1]. Patients with post-PCD empyema and pneumothorax were treated with another drainage tube, except one patient who underwent lung resection due to persistent BPF. The LoS was significantly shortened in the early PCD group (28.6 ± 25.5 vs. 39.3 ± 26.8 days, *p* = 0.045).

**Table 2:** Primary and secondary outcomes.

Outcome	Early PCD (*n* = 64)	Delayed PCD (*n* = 39)	*p*-value
*Primary outcome*			
90 days mortality	11 (17.2%)	10 (25.6%)	0.302
*Secondary outcomes*			
Length of hospital stay (days)^a^	28.6 ± 25.5	39.3 ± 26.8	0.045
*Complications*			
Bronchopleural fistula	1 (1.6%)	0 (0.0%)	1
Empyema	1 (1.6%)	3 (7.7%)	0.151
Hemoptysis	1 (1.6%)	0 (0.0%)	1
Pneumothorax	2 (3.1%)	1 (2.6%)	1

Except where indicated, data are numbers of participants, with percentages in parenthesis.

a Data are mean ± standard deviation.

### 3.3. Factors associated with 90 days mortality

Demographic characteristics and factors associated with 90 days mortality are summarized in Supplementary Table S2. Among 103 patients who received PCD for lung abscesses more than 3 cm in diameter, 82 (79.6%) recovered, and 21 (20.4%) had 90 days mortality. These two groups were similar in age, sex, and tube size. The 90 days mortality rate significantly increased with higher Charlson scores (7.8 ± 3.4 vs. 5.4 ± 3.2 in death and recovery group respectively, *p* = 0.006), secondary lung abscess (34.7% vs. 7.4% *p* = 0.001), and liver cirrhosis (50.0% vs. 17.2%, *p* = 0.014). The drainage duration (18.9 ± 29.6 vs.16.3 ± 12.9 days in recovery and death group respectively, *p* = 0.541), LoS (31.8 ± 27.4 vs. 35.8 ± 22.3 days in recovery and death group respectively, *p* = 0.497), complications (including BPF, empyema, hemoptysis, or pneumothorax), rationales of antibiotic use were similar between the two groups. There was a similar recovery rate between patients treated with empirical antibiotics and those treated with culture-based antibiotics (75.0% vs. 80.5%, respectively, *p* = 0.736).

### 3.4. Factors associated with LoS

Possible factors for the LoS are shown in Table 3. There was no significant difference in age, sex, and cause of lung abscess (primary or secondary). The patients with esophageal cancer (48.9 ± 42.8 vs. 30.7 ± 23.3 days respectively, *p* = 0.03), liver cirrhosis (48.8 ± 35.8 vs. 30.9 ± 24.8 days respectively, *p* = 0.041), longer duration of pre-drainage antibiotics (*p* < 0.001), positive sputum culture group (44.7 ± 33.4 vs. 22.5 ± 11.3 days respectively, *p* < 0.001), underwent subsequent surgery (50.9 ± 48.8 vs. 29.5 ± 19.1 days respectively, *p* = 0.003) had significant longer LoS. A mixed-effect model for the LoS is presented in Table 4. Early PCD significantly reduced the LoS in patients with lung abscesses [coefficient −10.52 (−19.69, −1.34), *p* = 0.025]. Positive sputum culture significantly increased the LoS [coefficient 19.35 (10.19, 28.50), *p* < 0.001].

**Table 3:** Analysis for possible factors for the length of hospital stay.

Variables	Length of hospital stay	*p*-value
Age (years)^a^	−0.093	0.348
Sex		0.39
Female (*n* = 24)	36.7 ± 32.0	
Male (*n* = 79)	31.4 ± 24.5	
Charlson comorbidity index^a^	−0.056	0.571
Cause		0.662
Primary (*n* = 54)	33.7 ± 27.4	
Secondary (*n* = 49)	31.4 ± 25.4	
DM		0.79
No (*n* = 68)	33.1 ± 30.0	
Yes (*n* = 35)	31.7 ± 17.8	
Solid tumor		0.844
No (*n* = 45)	32.0 ± 28.2	
Yes (*n* = 58)	33.1 ± 25.2	
Lung cancer		0.176
No (*n* = 76)	34.7 ± 28.6	
Yes (*n* = 27)	26.7 ± 17.9	
Esophageal cancer		0.03
No (*n* = 92)	30.7 ± 23.3	
Yes (*n* = 11)	48.9 ± 42.8	
Other cancer		0.8
No (*n* = 80)	32.3 ± 28.1	
Yes (*n* = 23)	33.9 ± 20.0	
CKD		0.134
No (*n* = 85)	30.8 ± 27.3	
Yes (*n* = 18)	41.1 ± 20.5	
Liver cirrhosis		0.041
No (*n* = 93)	30.9 ± 24.8	
Yes (*n* = 10)	48.8 ± 35.8	
Pre-procedure empyema		0.028
No (*n* = 74)	29.1 ± 19.3	
Yes (*n* = 29)	41.7 ± 38.1	
Pulmonary embolism		0.414
No (*n* = 95)	32.0 ± 27.2	
Yes (*n* = 8)	40.0 ± 12.2	
Duration of pre-drainage antibiotics (days)^a^	0.422	<0.001
Tube size (Fr.)^a^	−0.122	0.221
Distance of trans-aerated lung parenchyma		0.113
No (*n* = 91)	31.1 ± 23.2	
Yes (*n* = 12)	44.0 ± 43.8	
Drainage tube indwelling time (days)	0.031	0.755
Complications		
Bronchopleural fistula		0.839
No (*n* = 102)	32.6 ± 26.5	
Yes (*n* = 1)	38	
Empyema		0.313
No (*n* = 99)	32.1 ± 26.2	
Yes (*n* = 4)	45.8 ± 31.7	
Hemoptysis		0.084
No (*n* = 102)	32.2 ± 26.1	
Yes (*n* = 1)	78	
Pneumothorax		0.743
No (*n* = 100)	32.8 ± 26.7	
Yes (*n* = 3)	27.7 ± 15.0	
Positive abscess culture		0.274
No (*n* = 23)	27.3 ± 16.1	
Yes (*n* = 80)	34.2 ± 28.6	
Positive sputum culture		<0.001
No (*n* = 56)	22.5 ± 11.3	
Yes (*n* = 47)	44.7 ± 33.4	
Positive blood culture		0.236
No (*n* = 99)	33.3 ± 26.6	
Yes (*n* = 4)	17.3 ± 13.6	
Antibiotics		0.104
Empirical	22.8 ± 10.7	
Culture-based	34.5 ± 28.0	
Surgery		0.003
No (*n* = 88)	29.5 ± 19.1	
Yes (*n* = 15)	50.9 ± 48.8	
Lung resection		0.007
No (*n* = 94)	30.5 ± 23.2	
Yes (*n* = 9)	55.0 ± 44.7	
Decortication		0.115
No (*n* = 94)	31.4 ± 23.4	
Yes (*n* = 9)	45.9 ± 47.9	

Except where indicated, data are means ± standard deviations. ANOVA test was performed for categorical variables for the length of hospital stay.

a Correlations between continuous variables were analyzed using Pearson correlation.

**Table 4:** A mixed effect model for the length of hospital stay.

	Odds ratio	*p*-value
Esophageal cancer	8.05 (−7.58, 23.69)	0.309
Early PCD	−10.52 (−19.69, −1.34)	0.025
Pre-procedure empyema	8.89 (−1.40, 19.17)	0.090
Positive sputum culture	19.35 (10.19, 28.50)	<0.001
Lung resection	16.25 (−0.51, 33.02)	0.057
DM	−3.22 (−13.17, 6.74)	0.522
Liver cirrhosis	9.95 (−6.22, 26.12)	0.225

Data in parentheses are 95% CIs.

## 4. Discussion

Our results show that early PCD has a similar 90 days mortality and complication rate, but shorter LoS compared with delayed PCD. Our study’s overall recovery rate of patients who received PCD was 79.6%. Prior systemic review studies showed that malignancy-related abscesses and the occurrence of major complications were predictors of treatment failure (10). Our study had a similar finding: patients with secondary lung abscesses had a lower recovery rate than patients with primary lung abscesses (65.3% vs. 92.6%), which may relate to underlying disease. Besides, liver cirrhosis is associated with an increased mortality rate and prolonged LoS. Our drainage tubes ranged from 8 to 12 Fr., averaging 10 Fr.. There was no statistically significant difference between tube size and LoS (*p* = 0.221), possibly due to the small difference in tube diameter.

Although the traditional dogma that PCD for lung abscess was reserved for patients unresponsive to medical treatment, there is a paucity of data specifically comparing the timing of PCD and the outcome. Recent systematic review and meta-analysis showed no difference in outcomes or major complications between pre-PCD antibiotics in groups of more than 14 days and less than 14 days (10). Literature review of prior studies about PCD for lung abscess is summarized in Table 5. The drainage duration was longer in our study, which could be explained by our drainage management strategy. Prolonged drainage tube placement leads to adjacent pleural adhesions, thus lower the incidence of empyema or persistent BPF. Our study is the largest to date and is concordant with previous smaller studies that found that early PCD provides an equivalent outcome to delayed PCD.

**Table 5:** Summary of published studies on PCD for lung abscess.

Study	Case no.	Pre-procedure Abx <7 days	Pre-procedure Abx <14 days	Drainage duration (days)	Complications^a^
Rice et al. (15)	11	NA	NA	NA	0
Shim et al. (16)	5	0 (0.0)	3 (60%)	53	0
vanSonnenberg et al. (17)	19	NA	NA	9.8	1 (5.2%)
Ri et al. (18)	12	NA	NA	13.3	2 (16.7%)
Kim et al. (19)	12	8 (66.7%)	12 (100%)	9.9	1 (8.3%)
Prasad et al. (20)	12	0 (0)	11 (91.7%)	8	0
Jabłoński et al. (21)	9	NA	NA	NA	3 (33.3%)
Yunus et al. (22)	19	0 (0)	NA	NA	4 (21.1%)
Kelogrigoris et al. (23)	40	0 (0)	NA		5 (12.5%)
Gaballah et al. (14)	15	15 (100%)	15 (100%)	11.7	0
Matarese et al. (9)	8	0 (0)	0 (0)	7.7	1 (12.5%)
Our study	103	64 (62.1%)	NA	18.4	9 (8.7%)

The data presented are numbers of patients, with percentages in parentheses.

a Complications included BPF, empyema, pneumothorax, and hemoptysis. Abx, antibiotics; NA, not available.

Our relatively low overall complication rate of 8.7% in both groups may be explained by our procedure technique and imaging guidance method. We use the Seldinger method instead of the Trocar technique, and the procedure is performed using CT guidance. The Seldinger technique is considered safer because the puncture is made with a small-caliber needle, and thus the risk of adjacent structure injury in erroneous puncture is reduced. In addition, CT-guided puncture allows the clear visualization of the thorax during the procedure, free from overshadowing, compared with ultrasound guidance (24). A prior study suggested that traversal of normal lung parenchyma was a risk factor for complications like pneumothorax or hemothorax (10), but we could not confirm this in our study cohort.

Adequate adhesion around the abscess and drainage tube promotes spontaneous healing of bronchopleural fistula (BPF) without surgical intervention. Persistent BPF is the most dangerous and lethal complication of PCD. The treatment for BPF is difficult; surgical or bronchoscopic interventions was often required (25). In our studies, the majority of BPFs are sealed by early drainage and anti-infective treatment. Two reasons may explain our relatively low persistent BPF rate. First, in cases of empyema adjacent to an abscess, early placement of a pleural drainage tube is actively pursued to minimize the residual pleural space (26), and adequate adhesion around the abscess and drainage tube promotes the formation of a bronchopleurocutaneous fistula after 2 weeks of catheter placement and spontaneous healing of BPF without surgical intervention. Besides, the average size of the catheter was 10 Fr., which is smaller than the large bore chest tube. However, in a particular case with prolonged ventilator use, persistent severe air leakage under positive pressure breathing prevented effective adhesion in the surrounding costal cavity. Consequently, the patient necessitated a lobectomy to seal the BPF effectively.

Our patients received pre-drainage antibiotics with a mean duration of 7.9 ± 8.7 days. There is a tendency for patients in the recovery groups to have a lesser duration of pre-drainage antibiotics compared with the mortality group. Besides, the duration of pre-drainage antibiotics correlates with the LoS. Early PCD is beneficial for antibiotic stewardship. Our studies show that the positive rate of sputum culture was 45.6%, and blood culture was 3.9%. On the contrary, the positive rate from abscess culture is 77.7%. Although our results show similar mortality in patients who receive culture-based antibiotics and continued empirical broad-spectrum antibiotics, earlier pathogen detection allows antibiotics to be optimized. De-escalation of antibiotics can be performed earlier if a responsible pathogen is identified to avoid drug resistance.

The patients who received subsequent surgery have a comparable recovery rate to the overall recovery rate. Surgical treatment still plays an important role in lung abscesses. Of note, these patients are often operated on by open thoracotomy due to severe adhesion and unstable vital signs. The disadvantages of surgery include reduction of lung functional reserve after lung resection and intraoperative bleeding. Besides, residual dirty pleural space may be an infectious focus, and prolonged postoperative ventilator support may increase the risk of BPF (1, 27). Our results show that the timing of PCD placement (early vs. delayed) does not affect the risk of subsequent lung resection or decortication, nor influence the mortality rate. We suggest that for a lung abscess larger than 3 cm at initial diagnostic CT confirmed by radiological examination, the patient should receive early PCD to shorten recovery time.

Our surgical group utilizes both traditional thoracotomy and video-assisted thoracoscopic surgery (VATS). In our review of 15 surgical cases, 10 underwent VATS while 5 underwent open surgery. Thoracoscopy was introduced in our hospital in 2008. As VATS techniques matured, most surgeries gradually transitioned to VATS. The majority of VATS procedures involved mini-thoracotomy, which still required rib incision and the use of a rib spreader. True “complete” VATS remained relatively uncommon. In cases where patient condition was unstable, operators aimed to minimize operation time, but adhesive and undesirable lung deflation significantly prolonged the VATS completion time.

Our study has limitations. First, patients are not randomized to the early PCD and delayed PCD groups. The clinical physician made the decision on the timing of PCD insertion, which could lead to selection bias. However, the two groups have no significant difference in basic demographics or clinical characteristics. Second, this was a single-center retrospective study with a limited case number. However, it is the largest study to date. Third, our study does not compare with other treatment methods, such as direct surgery or medical treatment alone. However, medical treatment alone was less likely to cure lung abscesses more than 4 cm in diameter (15, 17). Fourth, our study had a long study period, the clinical management strategies might be different among physicians.

## 5. Conclusion

In conclusion, this cohort study demonstrates that early PCD within 1 week of diagnosis is associated with a shorter LoS for patients with liquefied lung abscesses larger than 3 cm. The 90 days mortality and complications were comparable between the early PCD and delayed PCD groups. These findings suggest the importance of emphasizing early drainage for lung abscesses exceeding 3 cm in clinical care paradigms and treatment guidelines.

## Data availability statement

The original contributions presented in the study are included in the article/Supplementary material, further inquiries can be directed to the corresponding author.

## Ethics statement

The studies involving human participants were reviewed and approved by National Cheng Kung University Hospital Institutional Review Board. Written informed consent for participation was not required for this study in accordance with the national legislation and the institutional requirements.

## Author contributions

P-CC and C-CC have full access to all the data in the study and take responsibility for the content of the manuscript. C-YL conceived and designed the study. P-CC, C-YL, and C-CC integrated data, analyzed the data, and wrote the manuscript. C-CC provided methodological and statistical support. C-YL and C-CC participated in editing of the manuscript. Y-CH, L-TH, T-JC, and Y-SL contributed to investigation and clinical inputs. All authors contributed to the article and approved the submitted version.

## Funding

This work is funded by the National Cheng Kung University Hospital of Taiwan (NCKUH-11201007 and NCKUH-11203049) and the Ministry of Science and Technology of Taiwan (MOST 111-2314-B-006-106).

## Conflict of interest

The authors declare that the research was conducted in the absence of any commercial or financial relationships that could be construed as a potential conflict of interest.

## Publisher’s note

All claims expressed in this article are solely those of the authors and do not necessarily represent those of their affiliated organizations, or those of the publisher, the editors and the reviewers. Any product that may be evaluated in this article, or claim that may be made by its manufacturer, is not guaranteed or endorsed by the publisher.
