# Supplementary material for: Early drainage reduces the length of hospital stay in patients with lung abscess
Source: Front Med (Lausanne). 2023 Sep 5;10:1206419. doi: 10.3389/fmed.2023.1206419 (PMC10508285; doi:10.3389/fmed.2023.1206419)
Supplement: Supplementary file 1 [file Table_1.DOCX]

Supplementary Material

Early drainage reduces the length of hospital stay in patients with lung abscess

Po-Chang Chiang, Chia-Ying Lin, Ya-Chun Hsu, Li-Ting Huang, Ta-Jung Chung, Yi-Sheng Liu, Chao-Chun Chang*

*** Correspondence:** Chao-Chun Chang: i5493149@gmail.com

# Supplementary Figures and Tables

**Supplementary Table 1. Baseline characteristics of patients undergoing PCD for lung abscess**

| Variables | Early PCD (n=64) | Delayed PCD (n=39) | *p* value |
| --- | --- | --- | --- |
| Age (y)* | 60.8 ± 18.9 | 59.8 ± 21.2 | 0.798 |
| Sex (male, %) | 51 (79.7%) | 28 (71.8%) | 0.358 |
| Height (cm) | 161 ± 17 | 159 ± 15.5 | 0.598 |
| BW (kg) | 56.3 ± 13.8 | 52.2 ± 13.4 | 0.185 |
| Size of abscess (cm)* | 7.5 ± 3.1 | 6.7 ± 2.4 | 0.199 |
| WBC_initial (x10^9^/L)* | 13.8 ± 8.0 | 13.8 ± 7.2 | 0.982 |
| WBC_3d (x10^9^/L)* | 11.7 ± 7.3 | 12.5 ± 8.3 | 0.641 |
| WBC_7d (x10^9^/L)* | 11.2 ± 7.3 | 13.1 ± 8.8 | 0.33 |
| CRP_initial (mg/dL)* | 210.4 ± 103.0 | 141.8 ± 81.5 | 0.068 |
| CRP_7d (mg/dL)* | 110.2 ± 103.3 | 72 ± 67.8 | 0.29 |
| Cause |  |  |  |
| Primary | 33 (51.6%) | 21 (53.8%) | 0.822 |
| Secondary | 31 (48.4%) | 18 (46.2%) |  |
| String-lock | 15 (24.2%) | 14 (37.8%) | 0.149 |
| Charlson comorbidity index* | 5.8 ± 3.2 | 6.0 ± 3.8 | 0.845 |
| Underlying disease |  |  |  |
| Solid tumor | 37 (57.8%) | 21 (53.8%) | 0.694 |
| Lung cancer | 19 (29.7%) | 8 (20.5%) | 0.304 |
| Esophageal cancer | 7 (10.9%) | 4 (10.3%) | 0.914 |
| Other cancer | 12 (18.8%) | 11 (28.2%) | 0.264 |
| DM | 24 (37.5%) | 11 (28.2%) | 0.334 |
| CKD | 8 (12.5%) | 10 (25.6%) | 0.088 |
| Liver cirrhosis | 6 (9.4%) | 4 (10.3%) | 1 |
| Pre-procedure empyema | 18 (28.1%) | 11 (28.2%) | 0.993 |
| Pulmonary embolism | 5 (7.8%) | 3 (7.7%) | 0.982 |
| Tube size (Fr.)* | 10.8 ± 2.7 | 10.2 ± 2.2 | 0.275 |
| Distance of trans-aerated lung parenchyma | 0.13 ± 0.41 | 0.09 ± 0.29 | 0.575 |
| Drainage tube indwelling time (d)* | 14.0 ± 15.6 | 25.6 ± 38.2 | 0.076 |
| Positive abscess culture | 53 (82.8%) | 27 (69.2%) | 0.108 |
| Positive sputum culture | 29 (45.3%) | 18 (46.2%) | 0.934 |
| Positive blood culture | 3 (4.7%) | 1 (2.6%) | 1 |
| Antibiotics |  |  | 0.597 |
| Empirical | 9 (14.1%) | 7 (17.9%) |  |
| Culture-based | 55 (85.9%) | 32 (82.1%) |  |
| Subsequent surgery |  |  |  |
| Lung resection | 6 (9.4%) | 3 (7.7%) | 1 |
| Decortication | 4 (6.3%) | 5 (12.8%) | 0.294 |
| Removal reason  Recovery  Surgery  Death  Dislodge  Poor function  Not removed | 36 (56.3%)  10 (15.6%)  4 (6.3%)  7 (10.9%)  5 (7.8%)  2 (3.1%) | 22 (56.4%)  3 (7.7%)  6 (15.4%)  6 (15.4%)  1 (2.6%)  1 (2.6%) | 0.435 |
| DM  No  Uncomplicated  End-organ damage | 40 (62.5%)  21 (32.8%)  3 (4.7%) | 28 (71.8%)  9 (23.1%)  2 (5.1%) | 0.573 |
| Hypertension  Solid tumor  No  Localized  Metastatic | 25 (39.1%)  27 (42.2%)  14 (21.9%)  23 (35.9%) | 12 (30.8%)  18 (46.2%)  6 (15.4%)  15 (38.5%) | 0.395  0.721 |
| Leukemia | 2 (3.1%) | 1 (2.6%) | 1 |
| Lymphoma | 0 (0%) | 0 (0%) |  |
| Moderate to severe CKD ^a^ | 8(12.5%) | 10(25.7%) | 0.084 |
| Myocardial infarction | 1 (1.6%) | 3 (7.7%) | 0.151 |
| Congestive heart failure | 1 (1.6%) | 2 (5.1%) | 0.555 |
| Peripheral vascular disease | 0 (0%) | 1 (2.6%) | 0.379 |
| CVA or TIA | 2 (3.1%) | 2 (5.1%) | 0.632 |
| Hemiplegia | 1 (1.6%) | 2 (5.1%) | 0.555 |
| Dementia | 3 (4.7%) | 1 (2.6%) | 1 |
| COPD | 7 (10.9%) | 4 (10.3%) | 1 |
| Connective tissue disease | 0 | 0 |  |
| Peptic ulcer disease | 10 (15.6%) | 9 (23.1%) | 0.344 |
| Liver disease ^b^  Mild  Mod to Severe | 5 (7.8%)  1 (1.6%) | 4 (10.3%)  0 (0.0%) | 0.678 |
| AIDS | 1 (1.6%) | 0 (0.0%) | 1 |
| Positive abscess culture |  |  |  |
| Klebsiella pneumoniae | 10 (15.6%) | 5 (12.8%) | 0.696 |
| Pseudomonas  Streptococcus anginosus | 8 (12.5%)  6 (9.4%) | 7 (17.9%)  1 (2.6%) | 0.447  0.249 |
| Aspergillosis | 1(1.6%) | 0 (0.0%) | 1 |
| Positive sputum culture |  |  |  |
| Klebsiella pneumoniae | 7 (10.9%) | 5 (12.8%) | 0.762 |
| Pseudomonas  Streptococcus | 9 (14.1%)  3 (4.7%) | 6 (15.4%)  2 (5.1%) | 0.854  1 |
| Aspergillosis | 2 (3.1%) | 0 (0.0%) | 0.525 |
| Positive blood culture |  |  |  |
| E. coli | 1 (1.6%) | 0 (0.0%) | 1 |
| Pseudomonas aeruginosa | 1 (1.6%) | 0 (0.0%) | 1 |
| Aspergillosis Ag positive | 0 (0.0%) | 1 (2.6%) | 0.379 |
| Antifungal treatment | 8 (12.5%) | 7 (17.9%) | 0.447 |

Note.—Except where indicated, data are numbers of participants, with percentages in parenthesis.

* Data are mean ± standard deviation

^a^ Severe = on dialysis, status post kidney transplant, uremia, moderate = creatinine >3 mg/dL (0.27 mmol/L)

^b^ Severe = cirrhosis and portal hypertension with variceal bleeding history, moderate = cirrhosis and portal hypertension but no variceal bleeding history, mild = chronic hepatitis (or cirrhosis without portal hypertension)

**Supplementary Table 2. Demographics, clinical characteristics and managements in recovery and death groups**

| Variables | Recovery (n=82) | Death (n=21) | *p* value |
| --- | --- | --- | --- |
| Age (y)* | 59.2 ± 20.7 | 65.3 ± 14.6 | 0.124 |
| Sex |  |  | 0.389 |
| Female | 21 (87.5%) | 3 (12.5%) |  |
| Male | 61 (77.2%) | 18 (22.8%) |  |
| Charlson comorbidity index | 5.4 ± 3.2 | 7.8 ± 3.4 | 0.006 |
| Cause |  |  | 0.001 |
| Primary | 50 (92.6%) | 4 (7.4%) |  |
| Secondary | 32 (65.3%) | 17 (34.7%) |  |
| DM |  |  | 0.336 |
| No | 56 (82.4%) | 12 (17.6%) |  |
| Yes | 26 (74.3%) | 9 (25.7%) |  |
| Solid tumor |  |  | 0.04 |
| No | 40 (88.9%) | 5 (11.1%) |  |
| Yes | 42 (72.4%) | 16 (27.6%) |  |
| Lung cancer |  |  | 0.012 |
| No | 65 (85.5%) | 11 (14.5%) |  |
| Yes | 17 (63.0%) | 10 (37.0%) |  |
| Esophageal cancer |  |  | 0.229 |
| No | 75 (81.5%) | 17 (18.5%) |  |
| Yes | 7 (63.6%) | 4 (36.4%) |  |
| Other cancer |  |  | 0.148 |
| No | 61 (76.3%) | 19 (23.8%) |  |
| Yes | 21 (91.3%) | 2 (8.7%) |  |
| CKD |  |  | 1 |
| No | 67 (78.8%) | 18 (21.2%) |  |
| Yes | 15 (83.3%) | 3 (16.7%) |  |
| Liver cirrhosis |  |  | 0.014 |
| No | 77 (82.8%) | 16 (17.2%) |  |
| Yes | 5 (50.0%) | 5 (50.0%) |  |
| Pre-procedure empyema |  |  | 0.554 |
| No | 60 (81.1%) | 14 (18.9%) |  |
| Yes | 22 (75.9%) | 7 (24.1%) |  |
| Pulmonary embolism |  |  | 1 |
| No | 75 (78.9%) | 20 (21.1%) |  |
| Yes | 7 (87.5%) | 1 (12.5%) |  |
| Duration of pre-drainage antibiotics (d) | 7.3 ± 8.2 | 10.3 ± 10.3 | 0.234 |
| Tube size (Fr.)* | 10.7 ± 2.7 | 9.9 ± 2.0 | 0.23 |
| Trans-aerated lung parenchyma |  |  | 0.706 |
| No | 73 (80.2%) | 18 (19.8%) |  |
| Yes | 9 (75.0%)) | 3 (25.0%) |  |
| Drainage tube indwelling time (d)* | 18.9 ± 29.6 | 16.3 ± 12.9 | 0.541 |
| Complications |  |  |  |
| Bronchopleural fistula |  |  | 1 |
| No | 81 (79.4%) | 21 (20.6%) |  |
| Yes | 1 (100%) | 0 (0.0%) |  |
| Empyema |  |  | 1 |
| No | 79 (79.8%) | 20 (20.2%) |  |
| Yes | 3 (75.0%) | 1 (25.0%) |  |
| Hemoptysis |  |  | 1 |
| No | 81 (79.4%) | 21 (20.6%) |  |
| Yes | 1 (100%) | 0 (0.0%) |  |
| Pneumothorax |  |  | 0.105 |
| No | 81 (81.0%) | 19 (19.0%) |  |
| Yes | 1 (33.3%) | 2 (66.7%) |  |
| Length of hospital stay (d)* | 31.8 ± 27.4 | 35.8 ± 22.3 | 0.497 |
| Positive abscess culture |  |  | 0.052 |
| No | 15 (65.2%) | 8 (34.8%) |  |
| Yes | 67 (83.8%) | 13 (16.3%) |  |
| Positive sputum culture |  |  | 0.235 |
| No | 47 (83.9%) | 9 (16.1%) |  |
| Yes | 35 (74.5%) | 12 (25.5%) |  |
| Positive blood culture |  |  | 0.184 |
| No | 80 (80.8%) | 19 (19.2%) |  |
| Yes | 2 (50.0%) | 2 (50.0%) |  |
| Antibiotics |  |  | 0.736 |
| Empirical | 12 (75.0%) | 4 (25.0%) |  |
| Culture-based | 70 (80.5%) | 17 (19.5%) |  |
| Surgery |  |  | 0.73 |
| No | 69 (78.4%) | 19 (21.6%) |  |
| Yes | 13 (86.7%) | 2 (13.3%) |  |
| Lung resection |  |  | 1 |
| No | 75 (79.8%) | 19 (20.2%) |  |
| Yes | 7 (77.8%) | 2 (22.2%) |  |
| Decortication |  |  | 0.682 |
| No | 74 (78.7%) | 20 (21.3%) |  |
| Yes | 8 (88.9%) | 1 (11.1%) |  |

Note.—Except where indicated, data are numbers with percentages in parenthesis.

* Data are mean ± standard deviation

Differences between categorical and continuous variables of recovery and death groups were compared using the Chi-square test or Fisher's exact test and Student t-test, respectively
